# The effects of SDF-1 combined application with VEGF on femoral distraction osteogenesis in rats

**DOI:** 10.1515/biol-2022-0851

**Published:** 2024-04-17

**Authors:** Fangang Fu, Mengqi Li, Shuye Yang, Gangqiang Du, Yingjiang Xu, Jianhao Jiang, Long Jia, Kai Zhang, Peng Li

**Affiliations:** Department of Orthopaedics, Yantai Affiliated Hospital of Binzhou Medical University, Yantai, China; Department of Orthopedics, Binzhou Medical University Hospital, Binzhou, 256603 China; Binzhou Medical University Hospital, Binzhou, China

**Keywords:** distraction osteogenesis, stromal cell-derived factor 1, vascular endothelial growth factor, bone marrow mesenchymal stem cells

## Abstract

Bone regeneration and mineralization can be achieved by means of distraction osteogenesis (DO). In the present study, we investigated the effect of stromal cell-derived factor 1 (SDF-1) and vascular endothelial growth factor (VEGF) on the new bone formation during DO in rats. Forty-eight Sprague–Dawley rats were randomized into four groups of 12 rats each. We established the left femoral DO model in rats and performed a mid-femoral osteotomy, which was fixed with an external fixator. DO was performed at 0.25 mm/12 h after an incubation period of 5 days. Distraction was continued for 10 days, resulting in a total of 5 mm of lengthening. After distraction, the solution was locally injected into the osteotomy site, once a day 1 ml for 1 week. One group received the solvent alone and served as the control, and the other three groups were treated with SDF-1, VEGF, and SDF-1with VEGF in an aqueous. Sequential X-ray radiographs were taken two weekly. The regeneration was monitored with the use of micro-CT analysis, mechanical testing, and histology. Radiographs showed accelerated regenerate ossification in the SDF-1, VEGF, and SDF-1 with the VEGF group, with a larger amount of new bone compared with the control group, especially SDF-1 with the VEGF group. Micro-CT analysis and biomechanical tests showed Continuous injection of the SDF-1, VEGF, and SDF-1 with VEGF during the consolidation period significantly increased bone mineral density bone volume, mechanical maximum loading, and bone mineralization of the regenerate. Similarly, the expression of osteogenic-specific genes, as determined by real-time polymerase chain reaction , was significantly higher in SDF-1 with the VEGF group than in the other groups. Histological examination revealed more new trabeculae in the distraction gap and more mature bone tissue for the SDF-1 with the VEGF group. SDF-1 and VEGF promote bone regeneration and mineralization during DO, and there is a synergistic effect between the SDF-1 and VEGF. It is possible to provide a new and feasible method to shorten the period of treatment of DO.

## 1 Introduction

Distraction osteogenesis (DO) is an important surgical technique that has been widely used in the treatment of bone defects caused by various causes [1]. One of the limitations of this technique is the long period of time required for the newly formed bone tissue to mineralize and consolidate [2]. Therefore, the need to shorten the treatment time has become a major issue for further expanding the application of DO. Shortening the total treatment time of DO is always by effectively promoting bone regeneration and remodelling in the distraction area. Several experimental approaches have been reported in the literature. These have included LMP-1gene transduction [3], mechanical stimulation, electrical stimulation [4], transplantation of mesenchymal stem cells [5] or hormones [6], and stimulation with Novel biodegradable biomaterials [7,8] and growth factors [9].

Bone regeneration and mineralization during DO is a sequential cascade. In the process, osteogenesis and angiogenesis are the two most important parts [10]. The formation of new bone in the DO process is closely related to the role of bone marrow mesenchymal stem cells (BMSCs) [11,12]. In addition, angiogenesis has been known to be critical for new bone formation. DO is to give appropriate external mechanical stimulation to the callus tissues in the distraction area and form a hypoxic and weakly acidic environment to promote the stromal cell-derived factor-1 (SDF-1) rapidly up-regulated. BMSCs express the chemokine receptor CXCR4 and BMSCs migration towards SDF-1 has been confirmed *in vitro* and *in vivo* [13]. The SDF-1/CXCR4 axis maintains Msc and likely other stem cells in the bone marrow and may play a crucial role in bone regeneration. Vascular endothelial growth factor (VEGF) and SDF-1 have been identified as key mediators of endothelial progenitor cell mobilization. Studies have shown that VEGF does not only stimulate angiogenesis during fracture repair but also has a direct effect on osteoblast differentiation [14]. Current research indicates that VEGF not only is the most important factor in promoting angiogenesis, but also stimulates endothelial cells to secrete osteo-anabolic factors, making VEGF an important multifunctional key factor during bone regeneration, far beyond its classical role as an angiogenic growth factor [15]. Richards et al. [16] established a rabbit bilateral tibial traction model to study the amount of new bone formation and the changes in bone tissue structure at the traction end and found that the fixation period was the main period for the formation of new bone during DO, while new bone formation was rare in the latent and traction periods. This study provided a basis for determining the time of biological and mechanical intervention in the process of DO. This may make a reasonable explanation for the effect of growth factors applied after the end of traction and entering the stationary phase may be more significant than that of the traction phase.

In the present study, we compared the effects of SDF-1 and VEGF applied during the consolidation phase of DO on bone regeneration and mineralization. To explore whether SDF-1 and VEGF have a synergistic effect on promoting bone regeneration and mineralization. It is hoped that our study will provide a feasible method to shorten the treatment time of DO.

## 2 Materials and methods

### 2.1 Animals

Forty-eight 8- to 10-week-old Sprague–Dawley rats with body weights of 350–380 g were used. Animals were acclimatized to local vivarium conditions at a temperature of 25–28°C and a humidity of 65% with free access to fresh clean water and a pelleted commercial diet. The animals were bred at the Institute for Clinical and Experimental Surgery, Jinan Pengyue Experimental Animal Breeding Company, China. This study followed the National guidelines for the care and use of animals and was approved by the Experimental Animal Ethics Committee of the Hospital of Binzhou Medical University(20191028). All surgeries were performed under anaesthesia, and efforts were made to minimize the suffering of the animals.


**Ethical approval**: The research related to animal use has been complied with all the relevant national regulations and institutional policies for the care and use of animals, and has been approved by the Experimental Animal Ethics Committee of the Hospital of Binzhou Medical University (20191028).

### 2.2 Surgical procedure

Before surgery, each animal were anaesthetised by an intraperitoneal injection of 2% pentobarbital sodium (40 mg/kg). Then, the rats were fixed on an ultraclean workbench, and the skin was prepared for operation. Four Kirschner wires with a diameter of 1.2 mm were inserted lateral to the femoral condyle at distances of 5, 10, 20, and 25 mm in an effort to keep the Kirschner wires parallel. The monolateral external distraction fixator (Tianjing Xinzhong Co., Tianjin, China) was adjusted to ensure suitability after the Kirschner wires were inserted. The external fixator was then removed, and mid-femur osteotomy was performed between the second and third Kirschner wires in the left femur of each animal. Surgical incisions were then sutured sequentially, and the femur was stabilised with a monolateral external distraction fixator. Postoperative pain was managed with a subcutaneous local infiltration injection of lidocaine after surgery. The surgical incision was observed daily after surgery, and penicillin (100,000 units/day) was administered intramuscularly for 3 days after the osteotomy. The suture was removed about 10 days after surgery. Each experimental rat was fed in a single cage after the operation. The skin around the site of the pin insertion was sterilized using alcohol to prevent infection.

Forty-eight Sprague–Dawley rats were randomized into four groups of 12 rats each. We established the left femoral DO model in rats. DO was performed at 0.25 mm/12 h after an incubation period of 5 days. Distraction was continued for 10 days, resulting in a total of 5 mm of lengthening. Based on previous reports in the literature [17,18], we formulated the concentration and frequency of injection pairs. After distraction, the solution was locally injected into the osteotomy site, once a day 1 ml for 1 week. One group received the solvent alone and served as the control, and the other three groups were treated with SDF-1, VEGF, and SDF-1 with VEGF in an aqueous. The control group was injected with 1 ml of PBS vehicle. The SDF-1 group was injected with 200 ng of SDF-1 in 1 ml of PBS solution; The VEGF group was injected with 50 ng of VEGF in 1 ml of PBS solution; The SDF-1with VEGF group was injected with 200 ng of SDF-1 and 50 ng of VEGF in 1 ml of PBS solution. Sequential X-ray radiographs were taken two weekly. The regenerate was monitored with the use of Micro-CT evaluation, mechanical testing, and histology.

### 2.3 Gross specimen observation

At 4 and 8 weeks after bone lengthening was stopped, three rats were randomly selected from each group. The left femur of each rat was dissected, and the bone healing mineralization in the distraction site was observed.

### 2.4 X-ray radiography evaluation

Radiography was performed on the 2nd, 4th, 6th, and 8th weeks after the cessation of distraction in all rats. Siemens X-ray radiographs were taken to assess osseous formation in the distraction area. The setting for the machine was 52 kV and 50 mA, with a 100-ms exposure time.

### 2.5 Microcomputed tomography (micro-CT) evaluation

Microstructural change within the distraction regenerate in the rat was quantitatively assessed using micro-CT(PerkinElmer Quantum GX II). On the 4th week after the cessation of distraction, all the rats were imaged using micro-CT at a custom isotropic resolution of 8-μm isometric voxel size with a voltage of 90 kV and a current of 200 μA. Bone mineral density (BMD) and bone volume (BV) of each specimen were recorded with the built-in software for analysis.

### 2.6 Real-time polymerase chain reaction (RT‒PCR)

Levels of osteoblast-related genes Runx-2, OPN, ALP, and OCN, were assessed using real-time PCR. The primers were synthesized by Shanghai Bioengineering Co., LTD. with 18S rRNA as an internal reference. The primers for Runx-2 were 5′-CCACCACTCACTACCACACG-3′ (sense) and 5′-GGACGCTGACGAAGTACCAT-3′ (antisense), for OPN were 5′-GATCGATAGTGCCGAGAAGC-3′ (sense) and 5′-TGAAACTCGTGGCTCTGATG-3′ (antisense), for ALP were 5′-GACAAGAAGCCCTTCACAGC-3′ (sense) and 5′-ACTGGGCCTGGTAGTTGTTG-3′ (antisense), for OCN were 5′-CCTGACTGCATTCTGCCTCT-3′ (sense) and 5′-AGGTAGCGCCGGAGTCTATT-3′ (antisense). The real-time PCR was performed with the aforementioned specific primers and a qPCR kit. All the results were confirmed by repeating the experiment three times.

### 2.7 Histology evaluation

On the 4th and 8th weeks after the cessation of distraction, three rats from each group were randomly selected. The femora were harvested for Histology evaluation. The specimens were then fixed in 10% neutral buffered formalin and were decalcified with 10% formic acid. Five-micrometer thick paraffin sections were prepared. Sections were stained with haematoxylin and eosin (H&E) to evaluate new bone formation.

### 2.8 Biomechanical evaluation

The femora were harvested for the micro-CT evaluation. After the micro-CT evaluation was completed, the mechanical properties of specimens were evaluated by a three-point bending test within 24 h after termination.

### 2.9 Statistics

All data are expressed as the mean ± standard deviation (*x* ± *s*). Data between groups were analysed by one-way analysis of variance, and means were compared in pairs. The statistical analysis was calculated by SPSS (version 22.0), and the level of significance was set at *P* < 0.05.

## 3 Results

### 3.1 Gross specimen observation

**Figure 1:**
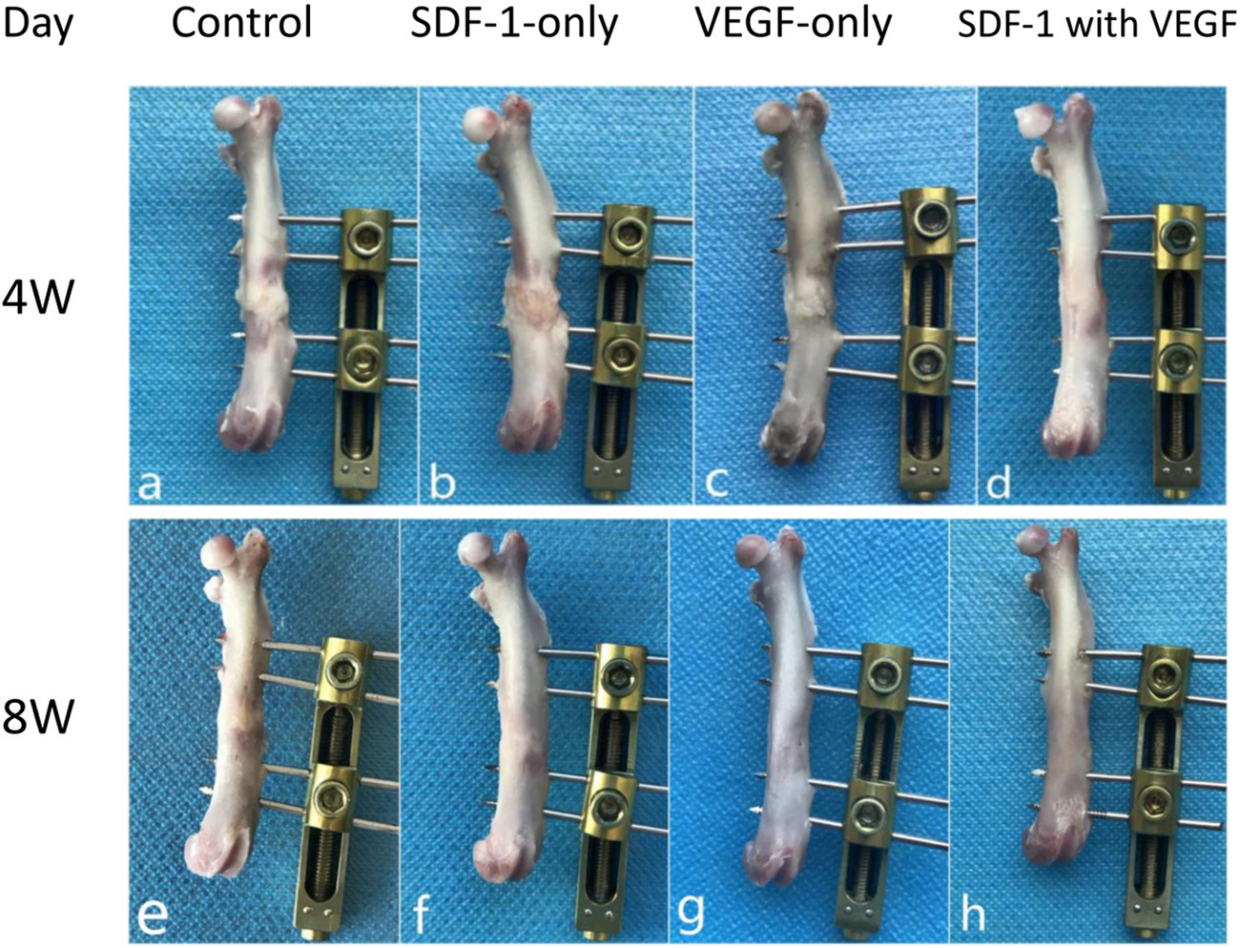
Gross specimen observation. At 4 weeks after the cessation of distraction (a–d), soft tissue could be easily observed in the distraction gap in the control group and VEGF group. In the SDF-1 group, osseous connections were mostly formed, and only a small amount of soft tissue was found on the surface. In SDF-1 with the VEGF group, an osseous connection was fully formed in the distraction gap, and the new bone had begun to reshape. On the 8th week after the cessation of distraction (e–h), the distraction gap had fully formed osseous connections in the control group, SDF-1 group, and VEGF group. In SDF-1 with the VEGF group, bone remodelling was completed, and the bone was closer to normal than that in the other three groups.

At 4 weeks after the cessation of distraction (a–d), soft tissue could be easily observed in the distraction gap in the control group and VEGF group. In the SDF-1 group, osseous connections were mostly formed, and only a small amount of soft tissue was found on the surface. In SDF-1 with VEGF group, an osseous connection was fully formed in the distraction gap, and the new bone had begun to reshape. On the 8th week after the cessation of distraction (e–h), the distraction gap had fully formed osseous connections in the control group, SDF-1 group, and VEGF group. In SDF-1 with the VEGF group, bone remodelling was completed, and the bone was closer to normal than that in the other groups (Figure 1).

### 3.2 X-ray analysis evaluation

**Figure 2:**
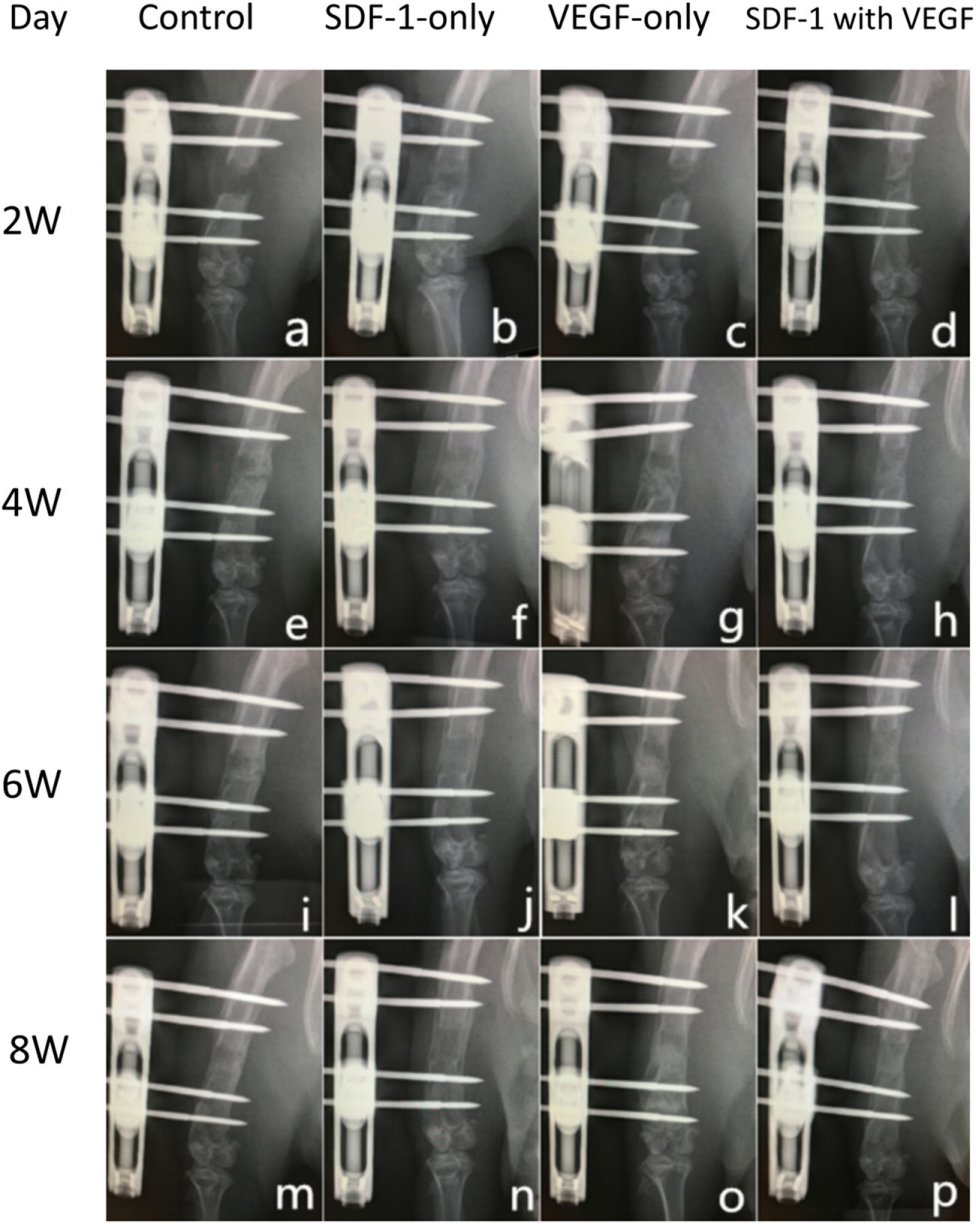
X-Ray evaluation. At 2 weeks after the cessation of distraction (a–d), none of the control group rat femurs showed a callus in the central zone of the distraction gap. There was a small amount of new bone formed in both the SDF-1 group and the VEGF group, and the formation of extra callus was more evident in the SDF-1 group than in the VEGF group. In SDF-1 with the VEGF group, new bone formation was apparent, and the stretching gap was reduced significantly. At 4 weeks after the cessation of distraction (e–h), there was obvious new bone formation in the control group, and the osteotomy line was clearly visible. The stretching gap in the VEGF group had almost disappeared, and the osteotomy line was obscure. In the SDF-1 group and SDF-1 with the VEGF group, the stretching gap exhibited bridging callus formation, but the density of callus in the SDF-1 group was lower than that of SDF-1 with the VEGF group. At 6 weeks after the cessation of distraction (i–l), the stretching gaps on rat femurs from the SDF-1 group, VEGF group, and SDF-1 with VEGF group demonstrated bone connection, whereas the osteotomy line on rat femurs from the control group still observed. The rat femurs from all groups exhibited bridging callus formation on the 8th week after the cessation of distraction (m–p), and the new bone remodelling from SDF-1 with the VEGF group was better than that from the other three groups.

**Figure 3:**
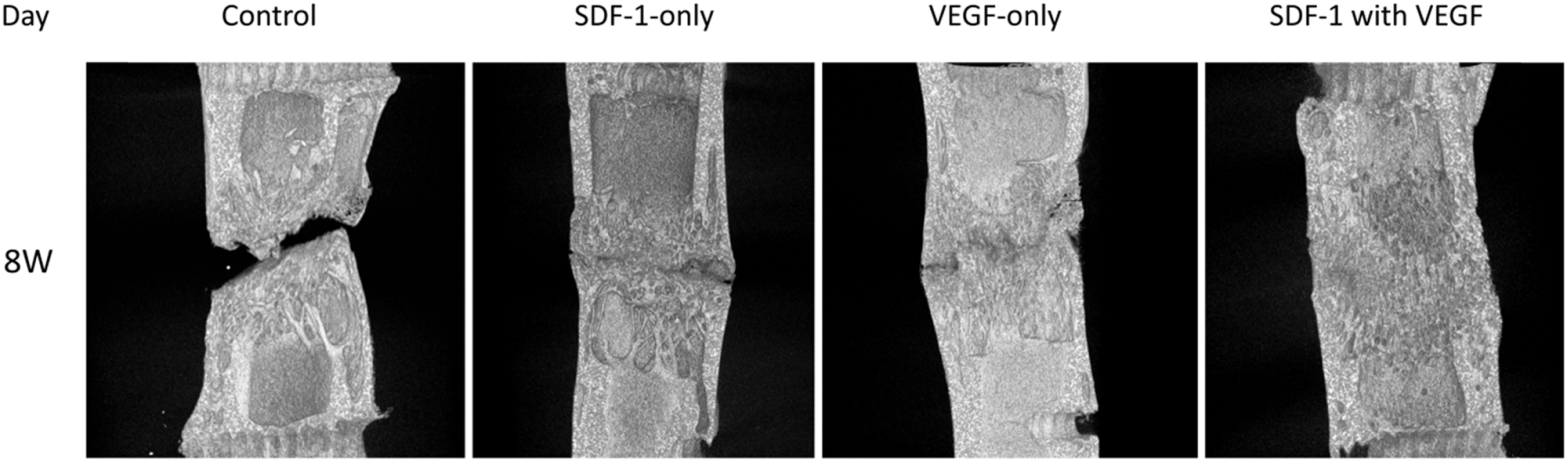
Micro-CT evaluation.

At 2 weeks after the cessation of distraction (a–d), none of the control group rat femurs showed a callus in the central zone of the distraction gap. There was a small amount of new bone formed in both the SDF-1 group and VEGF group, and the formation of extra callus was more evident in the SDF-1 group than in the VEGF group. In SDF-1 with the VEGF group, new bone formation was apparent, and the stretching gap was reduced significantly. At 4 weeks after the cessation of distraction (e–h), there was obvious new bone formation in the control group, and the osteotomy line was clearly visible. The stretching gap in the VEGF group had almost disappeared, and the osteotomy line was obscure. In the SDF-1 group and SDF-1 with the VEGF group, the stretching gap exhibited bridging callus formation, but the density of callus in SDF-1 group was lower than that of the SDF-1 with the VEGF group. At 6 weeks after the cessation of distraction (i–l), the stretching gaps on rat femurs from the SDF-1 group, VEGF group, and SDF-1 with VEGF group demonstrated bone connection, whereas the osteotomy line on rat femurs from the control group still observed. The rat femurs from all groups exhibited bridging callus formation on the 8th week after the cessation of distraction (m–p), and the new bone remodelling from SDF-1 with the VEGF group was better than that from the other three groups (Figures 2 and 3).

### 3.3 Micro-CT evaluation

**Figure 4:**
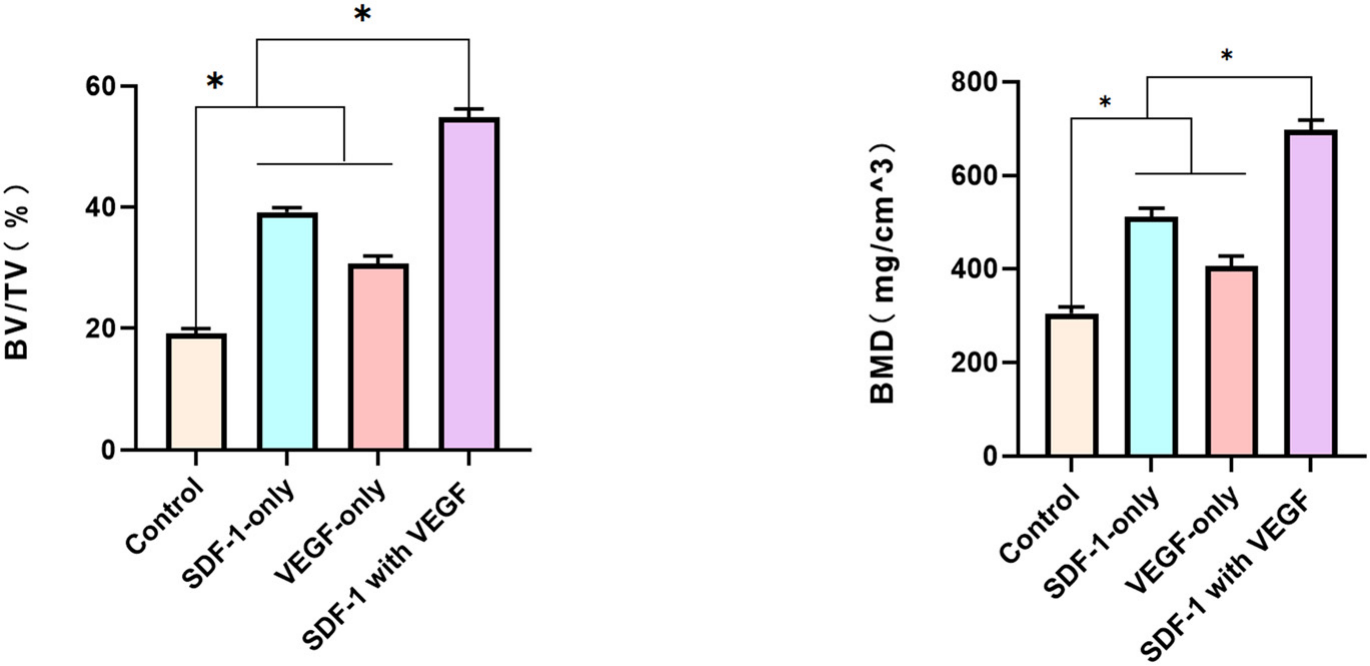
Micro-CT, bone formation and remodelling of the distraction regeneration in SDF-1 with VEGF group had greater values than the other groups at 4 weeks after the cessation of distraction.

Micro-CT, bone formation, and remodelling of the distraction regeneration in SDF-1 with the VEGF group had greater values than those of the other groups at 4 weeks after the cessation of distraction (Figure 4).

**Figure 5:**
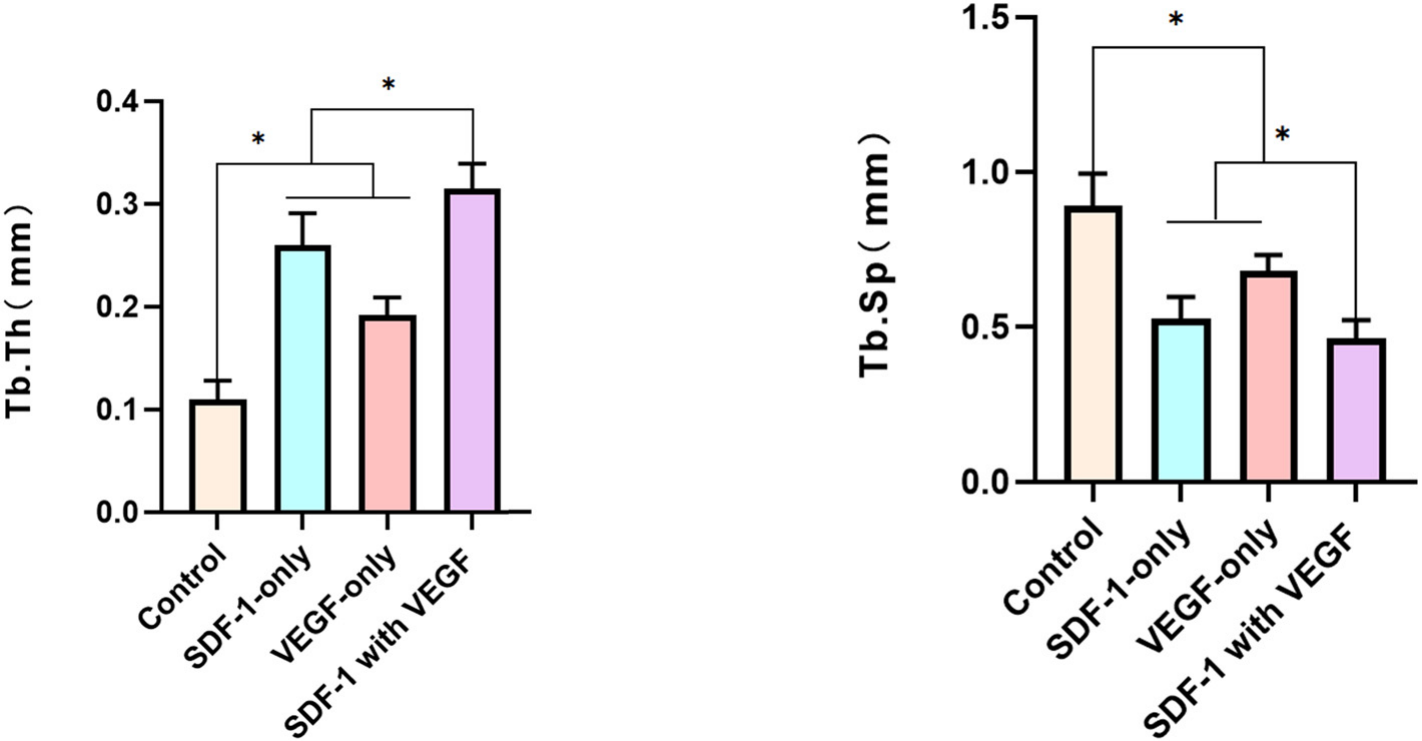
Two-dimensional quantitative analysis of distraction regeneration showed that the BV/tissue volume, BMD, Tb.Th, Tb.Sp in SDF-1 with the VEGF group had higher values than the other three groups (*P* < 0.05).

Two-dimensional quantitative analysis of distraction regeneration showed that the BV/tissue volume, BMD, Tb.Th, and Tb.Sp in SDF-1 with the VEGF group had higher values than those in the other three groups (*P* < 0.05 [Figure 5]).

### 3.4 RT‒PCR evaluation

**Figure 6:**
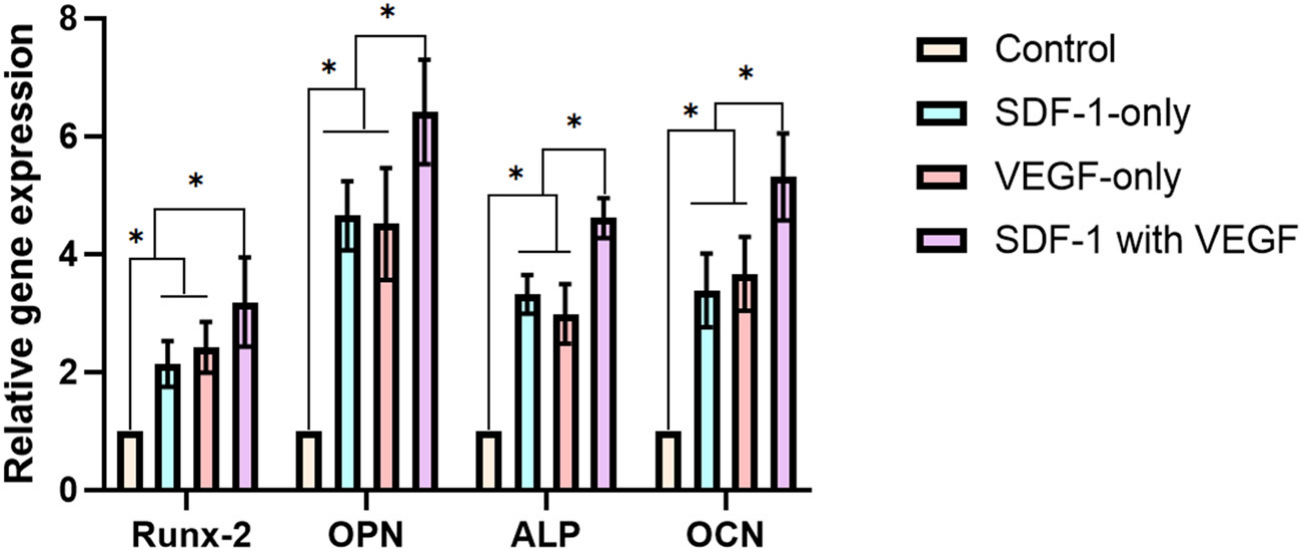
RT‒PCR evaluation. The expression levels of osteoblast-related genes, such as Runx-2, OPN, ALP, and OCN, in SDF-1 with the VEGF group were significantly higher than those in the other groups (*P* < 0.05). There was a significant difference between the SDF-1 group and the VEGF group compared with the control group (*P* < 0.05).

The expression levels of osteoblast-related genes, such as Runx-2, OPN, ALP, and OCN, in SDF-1 with the VEGF groups were significantly higher than those in the other groups (*P* < 0.05). There was a significant difference between the SDF-1 group and the VEGF group compared with the control group (*P* < 0.05 [Figure 6]).

### 3.5 Histology evaluation

**Figure 7:**
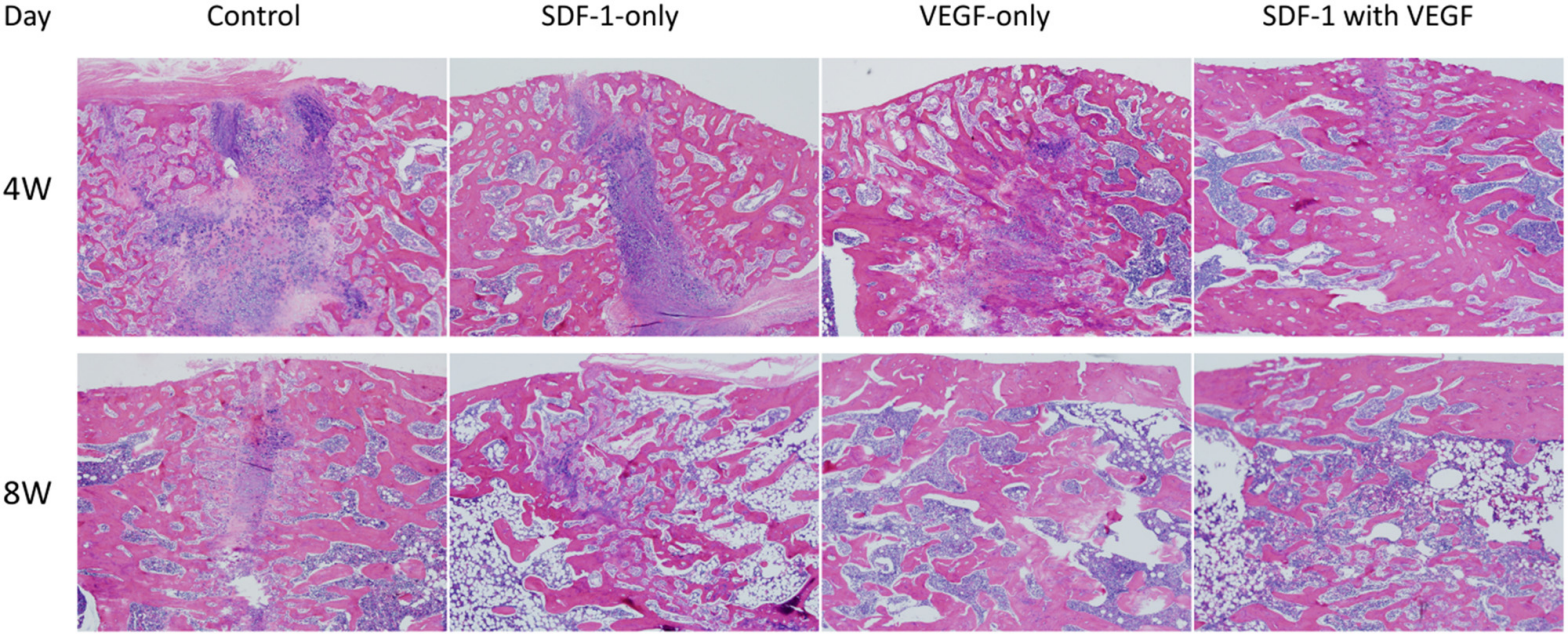
Histomorphometric evaluation. At 4 weeks after the cessation of distraction, H&E staining revealed abundant hypertrophic chondrocytes in the centre of the distraction gap in the control group, whereas in the centre of the distraction gaps in the SDF-1 group and the VEGF group, there was a small amount of fibrous and cartilage tissue, and the formed woven bone was larger than that in the control group. In SDF-1 with the VEGF group, the distraction gap was filled with newly woven bone. At 8 weeks after the cessation of distraction, the distraction gap in the control group mainly formed a bone connection; however, the number of bone trabeculae was less and of irregular arrangement. The number and volume of bone trabeculae in the SDF-1 group and VEGF group increased significantly compared with the control group. The bone trabeculae were arranged much more orderly in the SDF-1 group than in the VEGF group. The cortical bone was almost completely formed in SDF-1 with the VEGF group, and the medullary cavity was partially recanalised. The maturity of the new bone tissue was significantly better than that of the other three groups.

At 4 weeks after the cessation of distraction, H&E staining revealed abundant hypertrophic chondrocytes in the centre of the distraction gap in the control group, whereas in the centre of the distraction gaps in the SDF-1 group and VEGF group, there was a small amount of fibrous and cartilage tissue, and the formed woven bone was larger than that in the control group. In SDF-1 with the VEGF group, the distraction gap was filled with newly woven bone (Figure 7). At 8 weeks after the cessation of distraction, the distraction gap in the control group mainly formed a bone connection; however, the number of bone trabeculae was less and of irregular arrangement. The number and volume of bone trabeculae in the SDF-1 group and VEGF group increased significantly compared with those for the control group. The bone trabeculae were arranged much more orderly in the SDF-1 group than in the VEGF group. The cortical bone was almost completely formed in SDF-1 with the VEGF group, and the medullary cavity was partially recanalised. The maturity of the new bone tissue was significantly better than that of the other three groups (Figure 7).

### 3.6 Biomechanical evaluation

**Figure 8:**
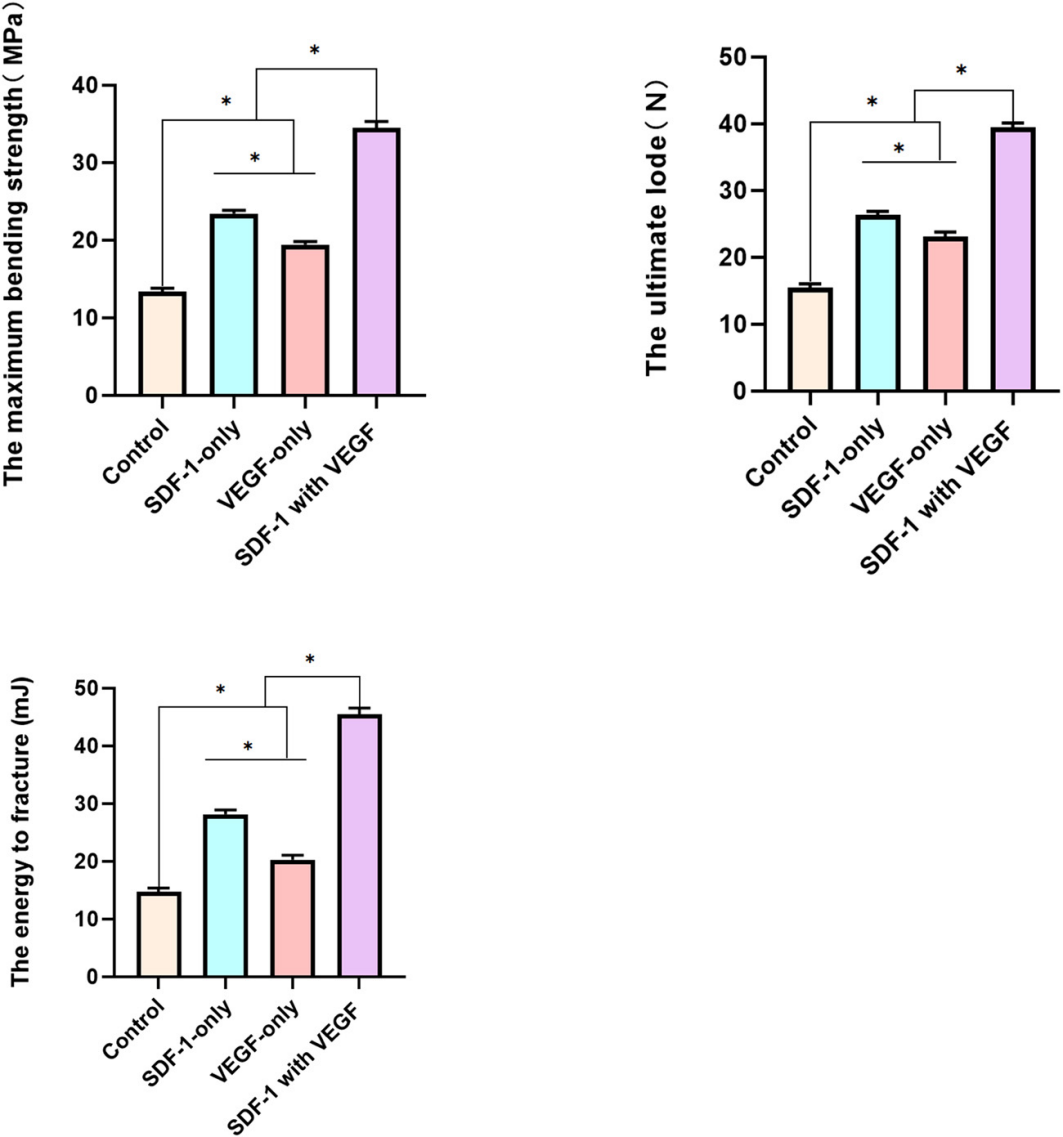
Biomechanical evaluation. At 4 weeks after the cessation of distraction, the femoral bending strength, ultimate load, and energy to fracture in the control group, VEGF group, SDF-1 group, and SDF-1 with VEGF group increased gradually, and the difference was statistically significant between any two groups (*P* < 0.05).

The femoral bending strength, ultimate lode, and energy to fracture in the control group, VEGF group, SDF-1 group, and SDF-1 with VEGF group increased gradually, and the difference was statistically significant between any two groups (*P* < 0.05 [Figure 8]).

## 4 Discussion

The present study is the first demonstration of the combined use of SDF-1 and VEGF in DO. Our results confirm the hypothesis that the combined use of SDF-1 and VEGF is capable of improving bone repair in the rat DO model. There are three phases of DO: the latency phase, the distraction phase, and the consolidation phase. Intramembrane osteogenesis is the main mechanism of osteogenesis in DO [19]. Not only a variety of osteogenic cells but also various cytokines are required for the newly formed callus in DO. Several growth factors, such as SDF-1, VEGF, BMP, TGF-β, IGF, and FGF, are expressed during the distraction period, promoting new bone formation in DO. In addition, they can accelerate the rate of bone mineralization and improve the quality of the new bone [20,21]. The number of identified growth factors continues to increase; therefore, intensive research on these factors could enable the development of minimally invasive or even non-invasive fracture treatments and provide new ideas and new solutions for the treatment of difficult diseases, such as non-union and bone defects.

SDF-1 is a chemotactic protein secreted by bone marrow stromal cells and endothelial cells. It is one of the major cytokines in the bone marrow, and it can effectively mobilise and recruit BMSCs to the distraction gap, induce their proliferation and differentiation into osteoblasts and chondrocytes, and promote new bone formation. It works mainly through the SDF-1/CXCR4 signalling pathway [22]. As reported, Xu et al. [23] used the rat tibia as a model for DO and injected the SDF-1/CXCR4 pathway inhibitor AMD3100 into the distraction gap. Their results showed that compared with the control group, the experimental group could significantly inhibit the formation of new bone and proved that SDF-1 could promote the formation of new bone effectively. After a latency period of 5 days, gradual distraction was performed at a rate of 0.25 mm per 12 h for 10 days, resulting in a total lengthening of 5 mm in this experiment. The pulling speed and frequency used in their experiments in rats obtained good results; thus, we also used the same pulling speed and frequency in our experiments. The expression of SDF-1 is regulated by hypoxia-inducible factor 1α (HIF-1α). Distraction causes tissue hypotension, leading to an increase in HIF-1α, followed by an increase in SDF-1 expression. CXCR4-positive progenitor cells (such as EPCs and BMSCs) are recruited to promote tissue regeneration through the SDF-1/CXCR4 signalling pathway [24]. In addition, SDF-1 mobilises and recruits vascular precursor cells to participate in angiogenesis and can promote the migration and proliferation of endothelial cells to initiate angiogenesis, which provides the necessary nutritional support for bone regeneration [25]. The SDF-1/CXCR4 signalling pathway is thought to be the key axis connecting EPCs, BMSCs, osteoblasts, and osteoclasts during bone regeneration and remodelling [26]. In our experiments, we also found that the group treated with SDF-1 had a larger callus, higher BMD, greater bending strength, and more mature bone tissue in the distraction gap compared with the groups without the application of SDF-1 within the same period. Once again, this proves the role of SDF-1 in promoting bone healing. These findings imply the feasibility of a new therapeutic approach to induce bone healing in DO by accelerating the homing and differentiation of osteoprogenitor cells.

VEGF possesses the function of angiogenesis, which is regulated by many factors, including growth factors, transcription factors, hormones, and mechanical stimulation. Hypoxia is considered a major driver of VEGF expression. Under hypoxic conditions, transcription factors and HIF-1α expressed by osteoblasts are significantly up-regulated, thereby promoting the synthesis of various angiogenic factors [27]. VEGF can not only promote the formation of new blood vessels and bring a variety of osteogenic cells, nutrients, oxygen, and minerals required for bone mineralization to the site of new bone formation but also regulate the proliferation, differentiation, and functional activity of osteoblasts and osteoclasts [10]. VEGF plays an important role in all phases of bone tissue repair and reconstruction, making it an important multifunctional key factor during bone regeneration far beyond its classical role as an angiogenic growth factor [28]. Ogilvie et al. [29] produced a mouse tibial non-union model. The test group and the control group were injected locally at the gap with VEGF and PBS solution, respectively, on Days 7, 8, and 9 after bone distraction. Tissue measurement was performed 27 days after the end of the distraction. The amount of new bone in the test group was significantly greater than that in the control group. Through this experiment, VEGF was proven to promote bone healing.

Research has shown that a certain amount of VEGF can accelerate the formation, mineralization, and remodelling of new bone; however, excessive VEGF may adversely affect bone regeneration. VEGF can regulate the differentiation and migration of osteoclasts; therefore, excess VEGF may recruit too many osteoclasts, resulting in excessive absorption of new bone [30]. In some experiments, VEGF was shown to have no significant effect on promoting bone healing. Peng et al. [31] conducted experiments using a mouse skull defect and found that when VEGF was injected alone into the defect area, a large amount of fibrous tissue was generated, without obvious bone formation. However, when they combined the applications of VEGF and BMP-4, they found that this had a synergistic effect in promoting bone healing. The combined application of the two factors was more significant than that of BMP-4 alone in promoting bone regeneration. This experiment showed that the combined application of VEGF and BMP-4 promoted the proliferation and differentiation of MSCs into chondroblasts. The formation of new bone was identified by intrachondral osteogenesis. Eckardt et al. [32] injected VEGF or VEGF inhibitors at the osteotomy area in a rabbit tibia osteogenesis test. Through measurements of blood flow, bone density, and biomechanics, it was shown that the application of VEGF or VEGF inhibitors had no significant effect on these indicators. Thus, they do not recommend the use of VEGF in DO to promote new bone formation. They believe that a large amount of endogenous VEGF has already been generated during the DO process. At this time, reapplication of exogenous VEGF may not have a significant promoting effect on bone regeneration; rather, excessive VEGF may adversely affect bone regeneration. Grosso et al. [33] found that osteo-angiogenic coupling is exquisitely dependent on VEGF dose, VEGF physiologically regulates both angiogenesis and osteogenesis, but its application in bone tissue engineering led to contradictory outcomes. It has been proved by research TFRD enhances angiogenicosteogenic coupling during DO by promoting type H vessel formation via PDGF-BB/PDGFR-β instead of HIF-1α/VEGF axis [34]. In our experimental study, we found that the application of exogenous VEGF has a certain effect on promoting bone healing, albeit its effect is weaker than that of SDF-1.

In our experiments, the blank control group formed bone connections in the distraction gap on the 6th week, and the SDF-1 application in combination with the VEGF group formed bone connections on the 4th week of the consolidation period. Our results also confirmed the view that the combined application of osteogenic and angiogenic factors has a synergistic effect on promoting bone healing in the process of DO, and it has a more significant effect than the application of one factor alone. We found that the application of SDF-1 or VEGF alone could promote bone healing; however, its effect was relatively limited. The combined application of VEGF and SDF-1 had a synergistic effect on promoting bone regeneration and could effectively shorten the treatment period of DO.

How did we determine when and how much VEGF and SDF-1 to use? Studies have shown that the expression of SDF-1 gradually increases, reaches a peak at the end of the distraction phase, and then gradually decreases. Cao et al. [24] conducted experiments using rats as animal models to compare the changes in SDF-1 in fracture and DO. Periodically, they estimated the content of SDF-1 in the new bone tissue in fracture and distraction regions by ELISA. Their results showed that in rats, the content of SDF-1 in both the fracture group and the DO group increased to approximately three times the normal value at Day 3 after osteotomy and then gradually decreased. However, the content of SDF-1 in the DO group increased again after the start of the distract phase, peaked at approximately five times the normal value at the end of the phase, and then decreased substantially during the first week of the fixed period to a normal level. A large number of DO experiments have shown that the expression of VEGF increases during the distract phase and gradually decreases during the fixed period [35,36]. Richards et al. [16] established a bilateral tibial DO model in rabbits to study the changes in the formation and structure of the new bone. The material was analysed at different time points, and the amount and structure of the new bone tissue were evaluated by micro-CT and histological examination. They found that the new bone began forming at the beginning of the consolidation period, and the rate of new bone increase was most significant from the end of the distraction phase until the 6th day of the consolidation period. The trabecular thickness and BV fraction increased significantly in the early stage of the consolidation period. This study provides a basis for determining the time of biological and mechanical interventions in the DO process. The consolidation period is the main period of new bone formation, while in the latency and active distraction periods, there is very little new bone formation. Therefore, the application of growth factors at the end of the distraction period in DO may have a more significant effect than their application during different periods. That is why we started injecting drugs when the distraction period was over.

Chen et al. [18] applied SDF-1 solutions with concentrations of 0, 100, 200, and 500 ng/ml in *in vitro* migration experiments of MSCs with SDF-1 and found that within a certain concentration range, the migration of MSCs was directly proportional to the concentration of SDF-1. The migration of MSCs did not increase significantly when the concentration of SDF-1 was higher than 200 ng/ml. Based on these findings, they proposed that the optimal concentration of SDF-1 to exert its largest chemotactic effect on MSCs was 200 ng/ml. Silva et al. [37] provided experimental evidence that the optimal concentration of VEGF to promote endothelial cell mitosis was 50 ng/ml. Using different concentrations of VEGF (3, 5, 10, 20, 30, 50, and 100 ng/ml) to observe its role in promoting endothelial mitosis, they found that the effect was concentration dependent. When the concentration of VEGF ranged between 5 and 20 ng/ml, there was no significant difference in promoting mitosis of endothelial cells, and when the concentration was equal to or higher than 50 ng/ml, its effect reached a plateau. Anderson et al. [38] performed endothelial cell migration experiments to prove that VEGF and SDF-1 can promote the migration of precursor and circulating endothelial cells and that the effect was stronger and significant when they combined the application of VEGF with SDF-1 (the concentrations used for VEGF and SDF-1 were 50 and 100 ng/ml, respectively). Street et al. [17] found that VEGF could directly enhance the activity of osteoblasts *in vitro*. When the VEGF concentration was between 25 and 50 ng/ml, the number of calcified nodules was the largest, and there was no significant difference between them. VEGF had the strongest ability to enhance alkaline phosphatase activity at 50 ng/ml. They found that topical application of VEGF could promote bone repair.

Our study showed that local application of SDF-1 and VEGF promotes bone regeneration and mineralization during DO, and there is a synergistic effect between SDF-1 and VEGF. It is possible to provide a new and feasible method to shorten the period of treatment of DO. The major limitation of this study is that the mechanisms underlying SDF-1 and VEGF promote bone regeneration and mineralization are not fully understood. In addition, growth factors have a short half-life and are easily degraded, requiring repeated injections. In the future, we will continue to conduct experiments using carriers to release growth factors to improve the utilization of growth factors. The complexity of the mechanics requires further investigation.
